# Laninamivir octanoate for post-exposure prophylaxis of influenza in household contacts: a randomized double blind placebo controlled trial

**DOI:** 10.1007/s10156-013-0622-9

**Published:** 2013-06-04

**Authors:** Seizaburo Kashiwagi, Akira Watanabe, Hideyuki Ikematsu, Shinichiro Awamura, Takako Okamoto, Mitsutoshi Uemori, Katsuyasu Ishida

**Affiliations:** 1 Kashiwagi Clinic, SS Building Hakata-Ekimae 4F, 3-21-15 Hakataekimae, Hakata-ku, Fukuoka, 812-0011 Japan; 2 Research Division for Development of Anti-Infective Agents, Institute of Development, Aging and Cancer, Tohoku University, Seiryomachi 4-1, Aoba-ku, Sendai, 980-8575 Japan; 3 Department of Clinical Trials, Center for Advanced Medical Innovation, Kyushu University, Fukuoka, Japan; 4 Clinical Development Department II, Daiichi Sankyo Co., Ltd., Hiromachi 1-2-58, Shinagawa-ku, Tokyo, 140-8710 Japan; 5 Clinical Data and Biostatistics Department, Daiichi Sankyo Co., Ltd., 1-2-58 Hiromachi, Shinagawa-ku, Tokyo, 140-8710 Japan

**Keywords:** Laninamivir, Neuraminidase inhibitor, Influenza, Prophylaxis, Post-exposure, Household contact

## Abstract

Laninamivir octanoate, a long-acting neuraminidase inhibitor, is an effective treatment for influenza. However, its effectiveness for the prevention of influenza has not yet been demonstrated. We conducted a double-blind, multicenter, randomized, placebo-controlled trial to determine whether laninamivir octanoate was superior to a placebo for post-exposure prophylaxis of influenza in household contacts. Eligible participants, who were household members who did not have influenza and were in contact with an influenza-infected index patient, were randomly assigned (1:1:1) to one of three groups: 20 mg of laninamivir octanoate once daily for 2 days (LO-2), 20 mg of laninamivir octanoate once daily for 3 days (LO-3), or a placebo. The primary endpoint was the proportion of participants who developed clinical influenza during a 10-day period. A total of 1711 participants were enrolled, and 1451 participants were included in the primary analysis. The proportion of participants with clinical influenza was 3.9 % (19/487) in the LO-2 group, 3.7 % (18/486) in the LO-3 group, and 16.9 % (81/478) in the placebo group (*P* < 0.001 for each of the laninamivir octanoate group). The relative risk reductions, compared with the placebo group, were 77.0 % [95 % confidence interval (CI) 62.7–85.8] and 78.1 % (95 % CI 64.1–86.7 %) for the LO-2 and LO-3 groups, respectively. The incidences of adverse events in the laninamivir octanoate groups were similar to that in the placebo group. The inhalation of 20 mg of laninamivir octanoate once daily for 2 or 3 days was well tolerated and effectively prevented the development of influenza in household contacts.

## Introduction

The family unit is a major source for the transmission of influenza viruses; the incidence of influenza in household contacts is higher than in the general population [1]. Although the primary means of influenza prevention is vaccination, anti-influenza drugs play an important role in preventing influenza among persons with a high risk of infection, such as household contacts [2], if a vaccine is not available, if exposure occurs before the vaccine has induced an immune response, or if there is no immune response to the vaccination. The efficacy of neuraminidase inhibitors, such as oseltamivir and zanamivir, for the prevention of influenza has been established [3–9].

Laninamivir potently inhibits the neuraminidase activities of various influenza A and B viruses, including subtypes N1–N9, influenza A(H1N1)pdm09 viruses, highly pathogenic avian influenza H5N1 viruses, and oseltamivir-resistant viruses [10, 11]. The efficacy of a single inhalation of laninamivir octanoate for influenza treatment in adults and children has been demonstrated [12–14]. In addition, studies in mice have shown the protective efficacy of the intranasal administration of laninamivir octanoate prior to virus infection [10].

We conducted a randomized placebo-controlled trial during the 2009 influenza pandemic season to evaluate the efficacy of the inhalation of 20 or 40 mg of laninamivir octanoate once a week (Days 1 and 8) for the post-exposure prophylaxis of influenza in household contacts during a 10-day period (unpublished data; registration number, JapicCTI-090941). The proportion of participants with clinical influenza, which was regarded as the primary endpoint, was 3.6 % (7/197) in the 20 mg group, 3.7 % (7/188) in the 40 mg group, and 6.6 % (12/183) in the placebo group. The protective efficacy was 45.8 % [95 % confidence interval (CI) −34.6 to 78.2 %] in the 20 mg group and 43.2 % (95 % CI −41.0 to 77.1 %) in the 40 mg group, and no significant difference was seen. However, in participants aged 10–19 years, among whom the transmissibility of the influenza A(H1N1)pdm09 virus was reportedly high, the protective efficacy seemed to be relatively higher.

Therefore, we conducted a trial to evaluate the efficacy of the inhalation of 20 mg of laninamivir octanoate once daily for 2 or 3 days for preventing the development of influenza in household contacts. In this trial, the laninamivir octanoate regimens were adjusted for a higher efficacy, since the prophylactic ability of laninamivir octanoate was insufficient at several days after the first dose of laninamivir octanoate in the previous trial.

## Patients and methods

### Trial design and population

This multicenter, double-blind, randomized, placebo-controlled trial was conducted between November 2011 and April 2012 at 80 primary care clinics in Japan. The trial was conducted in accordance with the Declaration of Helsinki and Good Clinical Practices [15]. The protocol was reviewed and approved by all applicable ethics and regulatory committees. All the index patients and the participants provided written informed consent.

Eligible participants were household members who had been in contact with the index patient within 48 h of symptom onset. An index patient was defined as someone who was infected with influenza A or B virus. The infection in the index patient was diagnosed using a rapid diagnostic kit. Participants were enrolled in the trial if they were found not to have influenza, had an axillary temperature of 36.9 °C or lower, had no influenza-like symptoms, and were at least 10 years old at the time of enrollment. The exclusion criteria were as follows: infection of other family members in addition to the index patient, an influenza vaccination, severe renal dysfunction, history of hypersensitivity to neuraminidase inhibitors, treatment with corticosteroid or other immunosuppressant, or treatment with a neuraminidase inhibitor within 4 weeks. Pregnant women, lactating women, and women who wished to become pregnant during the trial were also excluded in consideration of the safety of the participants and the next generation.

### Randomization and blinding

Participants were randomly assigned to one of the three treatments in a 1:1:1 ratio on an individual basis: The treatment groups were as follows: 20 mg of laninamivir octanoate administered once daily for 2 days (LO-2), 20 mg of laninamivir octanoate administered once daily for 3 days (LO-3), or a placebo. The LO-2 group was treated with 20 mg of laninamivir octanoate on Days 1 and 2 and with the placebo on Day 3. The LO-3 group was treated with 20 mg of laninamivir octanoate on Days 1, 2, and 3. The placebo group was treated with the placebo on Days 1, 2, and 3. Laninamivir octanoate or an identically packaged placebo, both containing lactose as the principal base, was administered by self-activated inhalation. A computer-generated block random allocation sequence was provided by Acronet Corporation (Tokyo, Japan) and was stratified according to the institution and whether the index patient was infected with influenza A or B virus. If the eligibility of the participant was confirmed, the investigator accessed the electronic data capture system and was notified of the allocation number of the test drug, which was individually packaged and numbered. The participants, index patients, investigators, and trial personnel were blinded to the group assignment throughout the trial.

### Trial procedures

The evaluation period for the study drug was set at 10 days in view of the duration of influenza virus excretion from the index patients and the incubation period of the influenza virus infection in the participants.

For all the index patients, anterior nose and posterior pharyngeal throat swabs were taken on Day 1 for the diagnosis of influenza. Influenza was screened using a rapid diagnostic kit and was confirmed using a laboratory virological test. The treatment of the index patients was unified to exclude any possible influence on the efficacy evaluation of the study drug. The index patients, except for teenagers, were treated with oseltamivir; teenagers were treated with zanamivir because the use of oseltamivir is not approved for use in that age group in Japan.

For all the participants, anterior nose and posterior pharyngeal throat swabs were taken on Days 1 and 11. When participants developed influenza-like symptoms during the trial duration (from Day 1 to Day 10), they were requested to visit the study site immediately and swabs were obtained for the confirmation of influenza infection. The participants who were diagnosed with influenza virus infection at the visits were provided with appropriate treatment and did not receive any subsequent doses of the test drugs. Participants recorded their axillary temperature and the severity of their influenza symptoms (headache, myalgia/arthralgia, fatigue, chills/sweats, nasal symptoms, sore throat, and cough) twice daily from Day 1 to Day 11. The severity of each influenza symptom was graded into four categories (0, absent; 1, mild; 2, moderate; 3, severe). For efficacy outcomes, the severity categories “2” and “3” were defined as the presence of an influenza symptom. Hematology, blood chemistry, and a urinalysis were performed on Days 1 and 11 for the safety assessment.

### Laboratory virological test

Each swab was placed in a sample tube containing viral transport medium and was transferred to a test laboratory. Influenza infection was confirmed by determining the influenza type and subtype based on a RT-PCR with specific primers designed from the hemagglutinin sequence of the influenza A(H1N1)pdm09, seasonal influenza A(H1N1), influenza A(H3N2), or influenza B viruses. The laboratory virological test was performed at Mitsubishi Chemical Medience (Tokyo, Japan).

### Efficacy outcomes

The primary endpoint was the proportion of participants who developed clinical influenza between Days 1 and 11. Clinical influenza was defined as the presence of laboratory-confirmed influenza, an axillary temperature of at least 37.5 °C, and at least two influenza symptoms [4]. The secondary endpoints were symptomatic influenza, asymptomatic influenza, and influenza infection. Symptomatic influenza, including clinical influenza, was defined as laboratory-confirmed influenza accompanied by an axillary temperature of at least 37.5 °C or at least one influenza symptom. Asymptomatic influenza was defined as laboratory-confirmed influenza accompanied by an axillary temperature of lower than 37.5 °C and no influenza symptoms. Influenza infection was defined as laboratory-confirmed influenza, regardless of the presence or absence of an axillary temperature of at least 37.5 °C or an influenza symptom.

### Statistical considerations

The primary population for evaluating efficacy was defined as participants in the full analysis set (FAS) who were confirmed to not be infected with the influenza virus on Day 1 and whose related index patient was confirmed to be positive for influenza virus on Day 1; such participants were designated as FAS index-infected virus-negative at baseline (FASIINAB). Additional analyses were conducted for FAS index-infected (FASII) and FAS participants. FASII was defined as participants in the FAS whose related index patient was confirmed to be positive for influenza virus on Day 1. The safety analysis included all the participants who received at least one dose of trial treatment and who underwent at least one safety assessment. All the analyses were performed using SAS^®^ System Release 9.2 (SAS Institute). All the reported *P* values were two-sided, and the level of significance was *P* < 0.05.

To test the trial hypothesis, we used the Fisher exact test to compare the proportion of participants who developed clinical influenza between each laninamivir octanoate group and the placebo group. Multiplicity was adjusted using the Hochberg method [16]. We also calculated the relative risk reduction compared with the placebo as the protective efficacy and the corresponding 95 % CI. We also analyzed symptomatic influenza, asymptomatic influenza, and influenza infection in the same manner as for the primary endpoint. Additionally, we explored the consistency of the treatment effect on the primary endpoint in prespecified subgroups.

The sample size was based on the assumptions that the protective efficacy of laninamivir octanoate would be at least 70 % and the proportion of participants who would experience clinical influenza during the trial would be 1.65 % for the laninamivir octanoate groups and 5.5 % for the placebo group, based on the previous prophylaxis trial of laninamivir octanoate conducted in 2009. On this basis, 470 participants in each group were required to achieve an 80 % power to detect the superiority of laninamivir octanoate over the placebo.

The trial was registered with JapicCTI (JapicCTI-111647).

## Results

### Trial population

**Fig. 1:**
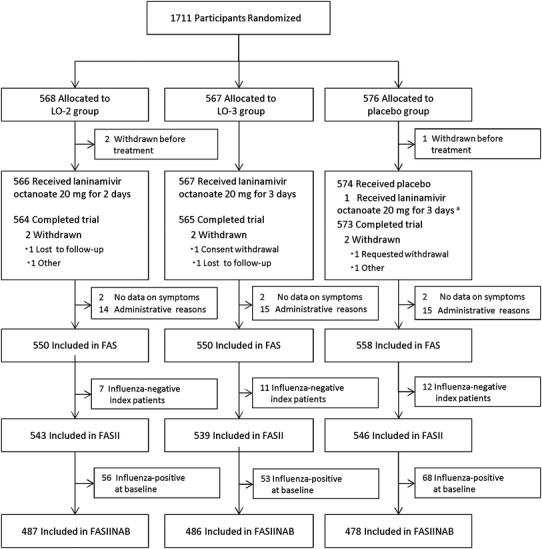
Participant flow chart. *LO-2* 20 mg of laninamivir octanoate administered once daily for 2 days; *LO-3* 20 mg of laninamivir octanoate administered once daily for 3 days; *FAS* the full analysis set, *FASII* the full analysis set index-infected, *FASIINAB* the full analysis set index-infected virus-negative at baseline. ^a^One participant who was allocated to the placebo group received 20 mg of laninamivir octanoate for 3 days. This participant was included in the originally allocated group in the full analysis set but was analyzed according to the actually administered treatment in the safety analysis set. A total of 1664 participants were included in the safety analysis set (552 participants in the LO-2 group, 553 in the LO-3 group, and 559 in the placebo group)

A total of 1711 participants were enrolled in the trial (Fig. 1). Of these, 47 participants were excluded from all analyses: three participants discontinued the trial before receiving any treatment, informed consent was not correctly obtained from two participants, and 42 participants treated at a trial center where the participation was halted because of issues related to the trial procedures and reliability of the data were excluded. Six other participants did not record their influenza symptoms and were excluded from the FAS. Of the FAS (1658 participants), 30 participants were excluded from the FASII because the related index patients were influenza virus-negative. In the FASII (1628 participants), 177 participants were influenza virus-positive at baseline and were excluded from the FASIINAB. A total of 1451 participants were included in the FASIINAB (487 participants in the LO-2 group, 486 in the LO-3 group, and 478 in the placebo group).

The baseline characteristics were well balanced among the three groups in the FASIINAB (Table 1), the FAS, and the FASII (data not shown). Most of the index patients were children under the age of 15 years, whereas most of the household contacts were the parents of index patients. Among the index patients, 91 % were infected with influenza A(H3N2) virus and 9 % were infected with influenza B virus (Table 1).

**Table 1:** Demographic and baseline characteristics of participants included in the full analysis set index-infected virus-negative at baseline

Characteristic	LO-2 (*N* = 487)	LO-3 (*N* = 486)	Placebo (*N* = 478)	Characteristic	LO-2 (*N* = 487)	LO-3 (*N* = 486)	Placebo (*N* = 478)
Participants				Index patients^a^			
Age				Age			
*Mean* *±* *SD* (*years*)	34.5 ± 9.7	33.8 ± 10.2	34.0 ± 9.2	*Mean* *±* *SD* (*years*)	7.5 ± 3.5	7.5 ± 4.3	7.7 ± 5.3
*Group* (*years*) [*no.* (*%*)]				*Group* (*years*) [*no.* (*%*)]			
10–14	43 (8.8)	58 (11.9)	43 (9.0)	0–4	98 (20.1)	96 (19.8)	89 (18.6)
15–19	18 (3.7)	9 (1.9)	11 (2.3)	5–9	264 (54.2)	281 (57.8)	275 (57.5)
20–29	36 (7.4)	37 (7.6)	42 (8.8)	10–14	116 (23.8)	97 (20.0)	99 (20.7)
30–39	241 (49.5)	235 (48.4)	264 (55.2)	15–19	6 (1.2)	5 (1.0)	7 (1.5)
40–49	144 (29.6)	141 (29.0)	113 (23.6)	20–29	3 (0.6)	4 (0.8)	3 (0.6)
50–59	4 (0.8)	5 (1.0)	4 (0.8)	30–39	0 (0.0)	2 (0.4)	2 (0.4)
60–	1 (0.2)	1 (0.2)	1 (0.2)	40–49	0 (0.0)	0 (0.0)	2 (0.4)
Sex [*no.* (%)]				50–59	0 (0.0)	1 (0.2)	0 (0.0)
Female	427 (87.7)	423 (87.0)	422 (88.3)	60–	0 (0.0)	0 (0.0)	1 (0.2)
Male	60 (12.3)	63 (13.0)	56 (11.7)	Sex [*no.* (%)]			
Time to first dose after onset in index patients				Female	238 (48.9)	236 (48.6)	219 (45.8)
*Mean* *±* *SD* (*h*)	21.6 ± 11.3	23.0 ± 12.5	22.5 ± 12.6	Male	249 (51.1)	250 (51.4)	259 (54.2)
*Group* (*h*) [*no.* (%)]				Rapid diagnostic test [*no.* (%)]			
0–12	101 (20.7)	101 (20.8)	99 (20.7)	Positive	487 (100.0)	486 (100.0)	478 (100.0)
12–24	207 (42.5)	181 (37.2)	189 (39.5)	Laboratory-confirmed influenza infection			
24–36	122 (25.1)	123 (25.3)	117 (24.5)	Virus Type and Subtype [*no.* (%)]			
36–48	54 (11.1)	80 (16.5)	70 (14.6)	2009H1N1	0 (0.0)	0 (0.0)	0 (0.0)
48–	3 (0.6)	1 (0.2)	3 (0.6)	H1N1	0 (0.0)	0 (0.0)	0 (0.0)
Relationship to index patient [*no.* (%)]				H3N2	443 (91.0)	440 (90.5)	434 (90.8)
Parent	423 (86.9)	413 (85.0)	415 (86.8)	B	43 (8.8)	44 (9.1)	43 (9.0)
Sibling	62 (12.7)	69 (14.2)	54 (11.3)	Mixed	1 (0.2)	2 (0.4)	1 (0.2)
Child	0 (0.0)	1 (0.2)	1 (0.2)	Treatment of influenza [*no.* (%)]			
Spouse	1 (0.2)	0 (0.0)	3 (0.6)	Oseltamivir	373 (76.6)	389 (80.0)	372 (77.8)
Other	1 (0.2)	3 (0.6)	5 (1.0)	Zanamivir	113 (23.2)	94 (19.3)	101 (21.1)
High-risk^b^ [*no.* (%)]	14 (2.9)	10 (2.1)	20 (4.2)	Other	1 (0.2)	3 (0.6)	5 (1.0)

*LO-2* 20 mg of laninamivir octanoate administered once daily for 2 days, *LO-3* 20 mg of laninamivir octanoate administered once daily for 3 days, *SD* standard deviation, *2009H1N1* influenza A(H1N1)pdm09, *H1N1* seasonal influenza A(H1N1), *H3N2* influenza A(H3N2), *B* influenza B

^a^More than one participant could be enrolled for each index patient. In this case, the index patient was counted once for each household contact who was enrolled. Actually, 1278 index patients (FASIINAB) were enrolled. In this table, the “N” is identical for household contacts and index patients in each treatment group. This is due to “reduplicative” counting

^b^Age ≥65 years or with concomitant illness (immunodeficiency, metabolic disorder, chronic respiratory illness, chronic renal impairment, or chronic heart disease)

### Efficacy

**Fig. 2:**
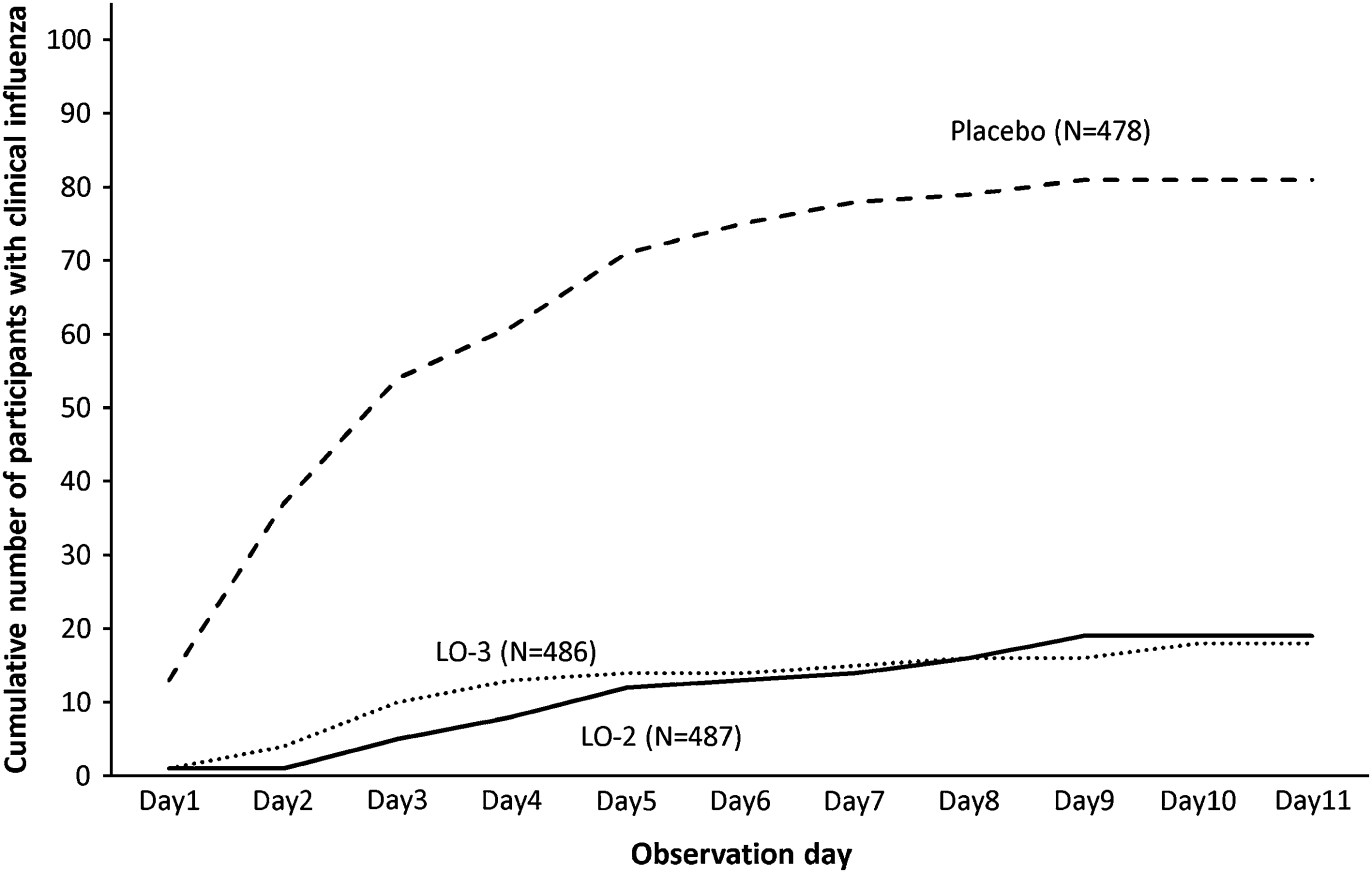
Cumulative number of participants with clinical influenza according to observation day in the full analysis set index-infected virus-negative at baseline. *LO-2* 20 mg of laninamivir octanoate administered once daily for 2 days, *LO-3* 20 mg of laninamivir octanoate administered once daily for 3 days

In the FASIINAB, the proportion of participants with clinical influenza, the primary endpoint, was 3.9 % (19/487), 3.7 % (18/486), and 16.9 % (81/478) in the LO-2, LO-3, and placebo groups, respectively (Table 2). Laninamivir octanoate significantly reduced the proportion of participants with clinical influenza, compared with the placebo (*P* < 0.001 for each laninamivir octanoate group). In this respect, no significant difference was observed between the LO-2 and the LO-3 groups. The protective efficacies were 77.0 % (95 % CI 62.7–85.8 %) and 78.1 % (95 % CI 64.1–86.7 %) in the LO-2 and LO-3 groups, respectively. Similar results were also obtained in the FAS and FASII (Table 2). In the placebo group, approximately 85 % (71/81) of the participants with clinical influenza developed influenza between Days 1 and 5, but the incidence appeared to decrease after Day 6. In contrast, laninamivir octanoate inhibited the development of clinical influenza throughout the trial period in each of the laninamivir octanoate groups (Fig. 2).

**Table 2:** Protective effects of laninamivir octanoate against influenza infection

Outcome	LO-2 (*N* = 487)			LO-3 (*N* = 486)			Placebo (*N* = 478)
	No./total (%)	*P* value^a^	Protective efficacy^b^ (95 %CI)	No./total (%)	*P*-value^a^	Protective efficacy^b^ (95 % CI)	No./total (%)
Primary endpoint (FASIINAB)							
Clinical influenza^c^	19/487 (3.9)	<0.001	77.0 (62.7 to 85.8)	18/486 (3.7)	<0.001	78.1 (64.1 to 86.7)	81/478 (16.9)
	–	–	–	–	1.00^d^	5.1 (−78.7 to 49.6)^d^	–
Secondary endpoints (FASIINAB)							
Symptomatic influenza^e^	33/487 (6.8)	<0.001	67.6 (53.0 to 77.7)	32/486 (6.6)	<0.001	68.5 (54.1 to 78.4)	100/478 (20.9)
Asymptomatic influenza^f^	17/487 (3.5)	0.41	24.2 (−41.0 to 59.2)	18/486 (3.7)	0.52	19.5 (−48.1 to 56.3)	22/478 (4.6)
Influenza infection^g^	50/487 (10.3)	<0.001	59.8 (45.5 to 70.3)	50/486 (10.3)	<0.001	59.7 (45.4 to 70.3)	122/478 (25.5)
Other endpoints							
Clinical influenza^c^ in FAS	29/550 (5.3)	<0.001	74.2 (61.9 to 82.5)	31/550 (5.6)	<0.001	72.4 (59.7 to 81.1)	114/558 (20.4)
Clinical influenza^c^ in FASII	29/543 (5.3)	<0.001	74.4 (62.2 to 82.7)	31/539 (5.8)	<0.001	72.5 (59.8 to 81.1)	114/546 (20.9)

*LO-2* 20 mg of laninamivir octanoate administered once daily for 2 days, *LO-3* 20 mg of laninamivir octanoate administered once daily for 3 days, *CI* confidence interval, *FASIINAB* the full analysis set index-infected virus-negative at baseline, *FAS* the full analysis set, *FASII* the full analysis set index-infected

^a^Analyzed using Fisher exact test

^b^Protective efficacy (relative risk reduction) = 100 × (1 − LO-2 or LO-3/Placebo)

^c^Clinical influenza was defined as the presence of laboratory-confirmed influenza, an axillary temperature of at least 37.5 °C, and at least two influenza symptoms

^d^Compared with LO-2

^e^Symptomatic influenza, including clinical influenza, was defined as laboratory-confirmed influenza accompanied by an axillary temperature of at least 37.5 °C or at least one influenza symptom

^f^Asymptomatic influenza was defined as laboratory-confirmed influenza accompanied by an axillary temperature of lower than 37.5 °C and no influenza symptoms

^g^Influenza infection was defined as laboratory-confirmed influenza, regardless of the presence or absence of an axillary temperature of at least 37.5 °C or an influenza symptom

The proportions of participants with symptomatic influenza were 6.8 % (33/487), 6.6 % (32/486), and 20.9 % (100/478) in the LO-2, LO-3, and placebo groups, respectively. The proportions of participants with influenza infection were 10.3 % (50/487), 10.3 % (50/486), and 25.5 % (122/478) in the LO-2, LO-3, and placebo groups, respectively. Laninamivir octanoate significantly reduced the proportions of participants with symptomatic influenza and the proportion of participants infected with influenza virus, compared with the placebo (*P* < 0.001 in each laninamivir octanoate group).

In the subgroup of participants whose related index patients were infected with the influenza A(H3N2) virus, laninamivir octanoate significantly reduced the development of clinical influenza, compared with the placebo (Table 3). The number of participants whose related index patients were infected with the influenza B virus was relatively small, and the trial did not have a sufficient statistical power to detect a significant difference. A similar trend for protective efficacy was generally seen for other subgroup categories examined in other prespecified subgroup analyses, except for the subgroup of participants aged 10–19 years in the LO-3 group (Table 3).

**Table 3:** Subgroup analyses for clinical influenza in the full analysis set index-infected virus-negative at baseline

Subgroup	LO-2			LO-3			Placebo
	No./total (%)	*P* value^a^	Protective efficacy^b^ (95 % CI)	No./total (%)	*P* value^a^	Protective efficacy^b^ (95 % CI)	No./total (%)
Virus type and subtype							
H3N2	16/443 (3.6)	<0.001	79.1 (64.7 to 87.6)	14/440 (3.2)	<0.001	81.6 (67.9 to 89.4)	75/434 (17.3)
B	3/43 (7.0)	0.48	50.0 (−87.1 to 86.6)	4/44 (9.1)	0.52	34.8 (−114.9 to 80.2)	6/43 (14.0)
Age (years)							
10–19	2/61 (3.3)	0.25	64.6 (−75.1 to 92.8)	7/67 (10.4)	1.00	−12.8 (−235.7 to 62.1)	5/54 (9.3)
20–29	1/36 (2.8)	0.03	85.4 (−11.1 to 98.1)	3/37 (8.1)	0.20	57.4 (−48.7 to 87.8)	8/42 (19.0)
30–39	12/241 (5.0)	<0.001	74.2 (52.8 to 85.9)	8/235 (3.4)	<0.001	82.4 (63.6 to 91.5)	51/264 (19.3)
40	4/149 (2.7)	<0.001	81.4 (46.1 to 93.6)	0/147 (0.0)	<0.001	100.0	17/118 (14.4)
Sex							
Female	17/427 (4.0)	<0.001	77.3 (62.2 to 86.4)	15/423 (3.5)	<0.001	79.8 (65.4 to 88.2)	74/422 (17.5)
Male	2/60 (3.3)	0.08	73.3 (−23.0 to 94.2)	3/63 (4.8)	0.18	61.9 (−40.3 to 89.7)	7/56 (12.5)
Time from onset of influenza in index patient to completion of first study treatment (h)							
<24	13/308 (4.2)	<0.001	73.0 (51.0 to 85.1)	13/282 (4.6)	<0.001	70.5 (46.5 to 83.7)	45/288 (15.6)
≥24	6/179 (3.4)	<0.001	82.3 (59.0 to 92.4)	5/204 (2.5)	<0.001	87.1 (67.7 to 94.8)	36/190 (18.9)
High-risk^c^	1/14 (7.1)	0.37	64.3 (−186.5 to 95.5)	0/10 (0.0)	0.27	100.0	4/20 (20.0)

*LO-2* 20 mg of laninamivir octanoate administered once daily for 2 days, *LO-3* 20 mg of laninamivir octanoate administered once daily for 3 days, *CI* confidence interval, *H3N2* influenza A(H3N2), *B* influenza B

^a^Analyzed using Fisher exact test

^b^Protective efficacy (relative risk reduction) = 100 × (1 − LO-2 or LO-3/Placebo)

^c^Age ≥65 years or with concomitant illness (immunodeficiency, metabolic disorder, chronic respiratory illness, chronic renal impairment, or chronic heart disease)

### Safety

Both laninamivir octanoate regimens were well tolerated. The most common adverse events were nasopharyngitis (2.2 % in the LO-2 group, 3.3 % in the LO-3 group, and 2.5 % in the placebo group) and upper respiratory tract inflammation (2.0 % in the LO-2 group, 1.3 % in the LO-3 group, and 0.9 % in the placebo group). The incidences of adverse events were 13.4 % (74/552), 13.0 % (72/553), and 11.6 % (65/559) in the LO-2, LO-3, and placebo groups, respectively. The incidence in each of the laninamivir octanoate group was similar to that in the placebo group. The incidences of adverse events considered by the investigator to be drug-related were 3.1 % (17/552), 4.7 % (26/553), and 2.7 % (15/559) in the LO-2, LO-3, and placebo groups, respectively. All the adverse events were regarded as being mild or moderate in severity. No deaths or other serious adverse events were reported.

## Discussion

This trial demonstrated that the inhalation of 20 mg of laninamivir octanoate once daily for 2 or 3 days was effective for preventing the development of influenza in household contacts. Laninamivir octanoate appears to be effective for preventing the transmission of influenza virus as well as the development of influenza illness, since laninamivir octanoate significantly reduced the proportion of participants with symptomatic influenza and the proportion of participants infected with influenza virus, compared with the placebo. In addition, the proportions of participants with clinical influenza were similar between the LO-2 and LO-3 groups, and inhalation of 20 mg of laninamivir octanoate once daily for 2 days was preferable for the lower dosing frequency.

The protective efficacy (LO-2, 77.0 %) after the administration of laninamivir octanoate once daily for only 2 days was comparable to that obtained using oseltamivir [5, 6] or zanamivir [8, 9]. This protective effect from fewer doses of laninamivir octanoate than oseltamivir or zanamivir can be explained by the pharmacokinetic characteristics of laninamivir octanoate, since a considerably high laninamivir concentration persisted in the lungs for 10 days after a single inhaled dose [17]. In the previous trial, the inhalation of laninamivir octanoate once a week (Days 1 and 8) seemed to be somewhat effective for post-exposure prophylaxis of influenza in household contacts, but the protective efficacy was not sufficient. However, in the present trial, the inhalation of laninamivir octanoate once daily for 2 days demonstrated a significant protective efficacy in household contacts. Thus, the second inhaled administration of laninamivir octanoate effectively contributed to the prevention of influenza in household members. The inhalation of laninamivir octanoate once daily for 2 days has the advantages of convenience and compliance over oseltamivir or zanamivir.

In this trial, we could not fully evaluate the efficacy for participants whose index patients were infected with influenza A(H1N1)pdm09, seasonal influenza A(H1N1), or influenza B viruses, since most of the index patients were infected with influenza A(H3N2) virus. Non-clinical study results have shown that laninamivir octanoate is effective against influenza A(H1N1)2009, seasonal influenza A(H1N1), and influenza B viruses [10, 11]. The prophylactic efficacy of laninamivir octanoate against these virus types should be further evaluated.

This trial excluded children younger than 10 years of age. However, considering the fact that a single inhaled dose of 20 or 40 mg of laninamivir octanoate was sufficient to treat children with seasonal influenza, including illnesses caused by oseltamivir-resistant viruses, and that the treatment was well-tolerated [13], laninamivir octanoate might be a valuable prophylactic agent against influenza for children. In addition, our trial excluded participants who came in contact with the index patients >48 h after the onset of illness. Postexposure chemoprophylaxis for persons should only be used when antivirals can be started within 48 h of the most recent exposure, as recommended by the Centers for Disease Control and Prevention [2, 18].

Generally, antiviral chemoprophylaxis should be considered for persons with a high risk of developing complications from influenza, such as the elderly (65 years or older), persons with complications (chronic respiratory illness, metabolic disorders including diabetes mellitus, chronic heart disease, or immunodeficiency), or pregnant women. Further research regarding the prophylactic administration of laninamivir octanoate in high-risk groups is needed.

In conclusion, the inhalation of 20 mg of laninamivir octanoate once daily for 2 or 3 days provided protection against influenza in household contacts. Our findings indicated that prophylaxis with laninamivir octanoate is an effective option for the post-exposure prophylaxis of influenza. Laninamivir octanoate is approved for the treatment of influenza and is widely used in clinical practice in Japan. In previous treatment trials [12–14] and post-marketing surveillance [19], no cases of bronchospasm or respiratory function deterioration have been reported, though laninamivir octanoate is an inhalant. Laninamivir octanoate appears to be a safe and useful agent for the prevention of influenza, as long as it is inhaled appropriately.

## Appendix: Lannimivir prophylaxis trial group

### Project Steering Committee

Seizaburo Kashiwagi (Kashiwagi Clinic, Fukuoka, Japan), Akira Watanabe (Research Division for Development of Anti-Infective Agents, Institute of Development, Aging and Cancer, Tohoku University, Sendai, Japan), Hideyuki Ikematsu (Department of Clinical Trials, Center for Advanced Medical Innovation, Kyushu University, Fukuoka, Japan), Katsuyasu Ishida, Shinichiro Awamura, Takako Okamoto, Keiya Murai, Soichiro Nishijima, Mitsutoshi Uemori (Daiichi Sankyo, Tokyo, Japan).

### Clinical Investigators who enrolled at least one patient

Toshiko Yamaguchi, Atsushi Shibasaki, Yutaka Igarashi, Hideki Sato, Takashi Kawashima, Masaaki Yoshihara, Kouta Saito, Tadahiko Ogasawara, Tomoyuki Shibuya, Shinya Enomoto, Eiki Oshika, Katsumi Yamada, Hisashi Murakawa, Nobuyuki Kaji, Hiromasa Noda, Haruo Kuroki, Ryohei Hisaki, Makoto Nonoda, Akiko Miyata, Daigo Fujii, Koji Maehara, Yoshihiko Maezawa, Minoru Nozaki, Hiroji Okawa, Yuko Miyazono, Yoshiyuki Morikawa, Hideto Kitagoh, Akiko Kurokawa, Takashi Abe, Ryuta Ono, Kenji Saito, Kazuhiko Muto, Jiro Takei, Mikiko Takano, Hideki Amemiya, Kazuo Arakawa, Shigenori Matsubara, Masaaki Kobayashi, Kazuki Hayakawa, Tadashi Matsuda, Toshihiko Sunami, Tooru Tsubota, Ryota Yoshimura, Toshikazu Takahashi, Yutaka Nakamura, Hiroshi Hirata, Keiichi Tsuboi, Masahiko Arita, Hidehisa Shinohara, Shigeru Onari, Michiko Tanabe, Hiroshi Taniguchi, Mikio Kihara, Tatsuo Yoshimitsu, Takashi Yokoyama, Yoshio Takasaki, Yuji Yamashita, Hiroshi Harada, Katsumaro Aida, Shizuo Shindo, Kunihisa Shimomura, Mitsuru Fukazawa, Yumi Kiyomatsu, Toru Umezu, Motohisa Ikeda, Minako Iwaya, Masaki Higashikawa, Takeshi Inamitsu, Yasuyuki Tokunaga, Satoru Okazaki, Yoichi Tokunaga, Makoto Ikezawa, Tetsunari Maeda, Shigeru Ikezawa, Toshimi Yoshimoto, Yoshiko Suginohara, Naoto Adachi, Hirofumi Tahara
